# Consumption of Resources (CR) in Critically Ill Patients (CIP) With Percutaneous Tracheostomy (PT)

**DOI:** 10.1186/2197-425X-3-S1-A482

**Published:** 2015-10-01

**Authors:** J Ruiz Moreno, E González Marín, MJ Esteve Paños, R Corcuera Romero de la Devesa, S Godayol Arias, MJ Riba Ribalta, N Conesa Folch, F Baigorri González, A Artigas Raventós

**Affiliations:** 1 Quirón Salud Hospital Universitario Sagrat Cor, Critical Care Department, Barcelona, Spain; 2 Quirón Salud Hospital Universitario Sagrat Cor, Emergency Department, Barcelona, Spain; 3 Hospital de Clínicas de Sabadell & Quirón Salud Hospital Universitario Sagrat Cor, Critical Care Department, Sabadell, Spain

## Introduction

It is considered that the CR of CIPs requiring PT is higher than the overall CIP population. However, both the identification of specific ICU procedures and the relative weight (RW) of the diagnostic related groups (DRG) case - mix system related to each CIP have not been researched sufficiently.

## Objectives

To identify and evaluate the CR of the CIPs with need of PT in comparison with the CIPs without requiring RRT.

To evaluate and compare the RW of the DRGs between CIPs with PT and without.

## Methods

Exclusion criteria: CIPs < 16 years, major burn patients, incomplete clinical documentation, and voluntary discharge

Variables analyzed:

Study: prospective, analytical, longitudinal, and observational

Period: January 1-2011 / June 30-2014 (42 months)

## Setting

Medical/Surgical ICU belonging to a 2790 acute care teaching hospital

Population: 2559 CIPs admitted consecutively to the ICU; sample: 53 CIPs

Exclusion criteria: CIPs < 16 years, major burn CIPs, incomplete clinical documentation, and voluntary discharge.

Variables analyzed:

a) length of stay (LOS), readmission

b) RW of DRG (AP-DRG 25.0 version)

c) invasive mechanical ventilation (IMV), non-invasive mechanical ventilation (nIMV)

d) renal replacement therapy (RRT)

e) Intracranial pressure, transcranial Doppler ultrasound

f) isolation measures

g) cardiac catheterization

Statistical analysis: Ji squared and contrast of means (Student's t)

## Results

## Conclusions

LOS and readmission are remarkably higher in the CIPs with PT

The RW of DRG is seven times higher in the CIPs with PT

Although expected, IMV and nIMV are also remarkably higher in the CIPs with PT

Isolation measures are more used in CIPs with PT (> 40 %)

RRT, ICP, TCDU and cardiac catheterization are more used in the CIPs with PT

Cardiac catheterization is more used in the CIPs without PT

**Table 1:** Results I

	Global	% or SD	PT	% or SD	no PT	% or SD	p value
CIPs	2559	100	53	2.1	2506	97.9	
Age	65.9	16.7	70.6	10.5	65.7	16.8	0.0005
Mortality	182	7.1	25	47.2	157	6.2	0.0001
LOS	3.51	6.5	34.9	19.0	2.85	3.8	0.0005
Readmission	136	5.3	8	15.1	128	5.1	0.0013
RW od DRG	4.2137	5	30.4955	15.3	3.6579	2.3	0.001

**Table 2:** Results II

	Global	% or SD	PT	% or SD	No PT	% or SD	p value
IMV	774	30.2	53	100	721	28.8	0.001
nIMV	298	11.6	31	58.5	267	10.6	0.001
RRT	91	3.6	12	22.6	79	3.1	0.001
ICP	14	0.5	3	5.7	11	0.4	0.001
TCDU	20	0.8	5	7.5	16	0.6	0.001
Isolation	92	3.6	23	43.4	69	2.7	0.001
Catheterization	103	4.0	0	0	103	4.1	0.001

## References

[1] Thomas AN, McGrath BA (2009). Patient safety incidents associated with airway devices in critical care: a review of reports to the UK National Patient Safety Agency. Anaesthesia.

[2] Silvester W, Goldsmith D, Uchino S, Bellomo R, Knight S, Seevanayagam S (2006). Percutaneous versus surgical tracheostomy: a randomised controlled study with long term follow up. Crit Care Med.

